# Sudden Deaths in Laryngeal Disease

**Published:** 1890-07-19

**Authors:** 


### SUDDEN DEATHS IN LARYNGEAL DISEASE.

Dr. Botey, of Barcelona, one of the most distinguished physicians in Spain, describes several instances of sudden death occurring in the course of chronic laryngeal disease, the deaths being due to a variety of causes, but some taking place under circumstances, *e.g.*, shortly after a laryngoscopic examination, calculated to suggest undeserved reflections on the procedure of the medical attendant, and against which it is well to be forewarned and forearmed.

(1) A man, aged 45, had for a year suffered from hoarseness and difficulty in deglutition. A laryngoscopic examination showed paralysis or retraction of one vocal cord, compensated in phonation by the other being carried over the middle line. Further examination gave negative results. During the introduction of a mirror the patient was seized with hæmorrhage, and died after having lost over two litres from the rupture of an aneurism of the aorta into the trachea.

(2) The next case was that of a man aged 62, who suffered from severe dyspnœa and symptoms of stenosis of the larynx. The laryngoscope left no doubt as to the presence of carcinoma, and a large pedunculated tumour was seen nearly closing the glottis, but an attempt to remove it had to be abandoned. The patient died suddenly a few hours later.

(3) A third suffering from tubercular ulceration of the lower lip, tongue, pharynx and larynx, as well as tuberculosis of both lungs, but whose larynx had not recently been examined, died while in the act of writing a letter.

(4) The fourth patient, aged 43, in whose case the laryngoscope revealed unmistakable carcinoma of the right arytænoid cartilage, with enlargement of the glands of the neck of six months' duration, died suddenly long after the last examination, although there was no trace of dyspnœa or of dysphagia.

Botey thinks that in most of these cases of unexpected death the pressure exerted by the enlarged peritracheal glands on the recurrent nerves causes paralysis of the glottis.

